# Short-term psychotherapeutic treatment in adolescents engaging in non-suicidal self-injury: a randomized controlled trial

**DOI:** 10.1186/1745-6215-14-294

**Published:** 2013-09-13

**Authors:** Gloria Fischer, Romuald Brunner, Peter Parzer, Franz Resch, Michael Kaess

**Affiliations:** 1 Section for Disorders of Personality Development, Clinic of Child and Adolescent Psychiatry, Centre of Psychosocial Medicine, University of Heidelberg, Blumenstrasse 8, Heidelberg 69115, Germany

**Keywords:** Non-suicidal self-injury, Self-harm, Randomized controlled trial, The Cutting-Down Programme, Adolescents, Short-term therapy

## Abstract

**Background:**

Worldwide, prevalence rates of adolescent non-suicidal self-injury (NSSI) range between 13 and 45%. In Germany, lifetime prevalence of NSSI is around 25% in non-clinical samples, and the one-year prevalence for repetitive NSSI is 4%. NSSI is present in the context of several axis I and II disorders (for example, affective disorders or borderline personality disorder); however, preliminary evidence suggests that it would be justified to consider NSSI as its own diagnostic category. Despite the large impact of this behavior, there is still a lack of evidence-based, specific, and effective manualized treatment approaches for adolescents with NSSI.

**Methods/Design:**

The study is designed as a randomized controlled trial (RCT) to test the effectiveness of a new cognitive-behavioral treatment manual for self-harming adolescents - the ‘Cutting-Down-Programme’ (CDP). A total of 80 adolescents aged between 12 and 17 years from a region in Southern Germany who have engaged in repetitive NSSI (≥5 incidents) in the last 6 months will be randomized into a treatment group (CDP) or a control group that will receive treatment as usual (TAU). The adolescents will be assessed by means of structured interviews and questionnaires at three time points (before treatment, directly after treatment and six months after treatment). Primary outcome criterion is a significant reduction (or remission) in the frequency of NSSI. Secondary outcome criteria are depressivity as well as general well-being and self-worth. Additionally, comorbid psychiatric disorders and childhood adversity will be evaluated as predictors of therapeutic outcome.

**Discussion:**

Recently, a pilot study in the United Kingdom showed significant reductions in self-harming behavior, depressive symptoms and trait anxiety. This is the first RCT to test the effectiveness of a short-term psychotherapeutic intervention in outpatients engaging in NSSI.

**Trial registration:**

The study is registered in the German Clinical Trials Register DRKS00003605.

## Background

Non-suicidal self-injury (NSSI) can be defined as a self-inflicted, deliberate, direct destruction of body tissue without conscious suicidal intent, which is not socially accepted [1,2]. During adolescence, prevalence ranges between 13 and 45% worldwide [1,3,4], and studies in the same population in Germany suggest a similar prevalence [4,5]. In a large school-based, cross-sectional study conducted during 2004/2005 in the defined catchment area (southwestern Germany), 15% of the 15-year-old adolescents included reported NSSI in the previous six months; 4% of them even reported repetitive NSSI [5]. Another study within the same geographical area from 2011 revealed a lifetime prevalence of any incident of NSSI of 35% among 14- to 16-year-old adolescents [6].

NSSI is often accompanied by a variety of psychological problems and disorders, but until recently, borderline personality disorder (BPD) was the only disorder in the classification systems (Diagnostic and Statistical Manual of Mental Disorders-IV (DSM-IV) and International Statistical Classification of Diseases and Related Health Problems-10 (ICD-10) that included self-injury as a diagnostic criterion. NSSI can occur in the context of depressive disorders, eating disorders, anxiety disorders, conduct disorders, substance-use disorders and post-traumatic stress disorders [7,8]. In addition, NSSI is highly associated with suicide attempts and death by suicide in adolescents [5,9]. As a result of increasing knowledge on the phenomenon of NSSI and its growing importance, NSSI has now been included as a section 3 disorder in the new DSM-5 [10-12]. This fact underscores the need for further research concerning NSSI and for the development of specific therapeutic concepts that are empirically validated for the condition.

To our knowledge, specific therapeutic programs focusing specifically on NSSI are rare [10]. The only existing manuals were originally developed for treatment of BPD, and consequently treat NSSI as one symptom of this disorder [13,14]. In this context, the Dialectic Behavioral Therapy for Adolescents (DBT-A) is the most common program [15], but its effectiveness in reducing NSSI has not been proven in a randomized controlled trial (RCT) so far [16]. Additionally, extensive therapeutic approaches like those for BPD may not always be adequate in light of the high prevalence of adolescent NSSI. A new therapeutic approach using mentalization-based therapy (MBT) for self-harming adolescents was recently published by Rossouw and Fonagy. In their RCT, they were able to achieve a recovery rate of 44% in the MBT group compared to 17% in the group receiving treatment as usual (TAU) [17]. However, length of treatment during this trial was comparable to psychotherapeutic interventions such as DBT-A. Treatment of NSSI is indispensable and, therefore, there is a great need for specific and effective psychotherapeutic treatment programs for adolescent NSSI. Such a program should focus on NSSI itself and needs to be time and cost efficient [18]. It should aim to reduce distress and impairment associated with negative emotional states in young people so as to decrease subsequent NSSI [16].

Evans *et al*. [19] at Kings College London, UK, developed a short-term therapy for adults with deliberate self-harm (DSH, which includes suicidal behavior and NSSI). This cognitive-orientated and problem-focused short-term therapy program, called the ‘Manual-Assisted Cognitive-behavioural Therapy’ (MACT), consists of a maximum of six therapeutic sessions and is assisted by a detailed manual. The content of MACT involves problem finding and solving, advantages and disadvantages of DSH, basic cognitive techniques to manage emotions and negative thinking, finding coping strategies and relapse prevention. In a RCT, the MACT program was tested versus TAU in 36 patients following episodes of DSH. The patients in the TAU group were followed by psychiatrists, social workers, members of the community mental health team or nobody. Most patients in the MACT group had therapeutic sessions conducted by psychiatrists, nurses or social workers, but a few of them (22.2%) only had the booklet, which they worked through by themselves. As a result, attempted suicide, the frequency of self-harm episodes and the number of depressive symptoms were significantly lower in the MACT treatment group than in the TAU group [19].

Taylor *et al*. [20] adapted the MACT for adolescents and tested it in an initial study in England. The adolescent version of the program is called the ‘The Cutting-Down Programme’ (CDP) and consists of 8 to 12 individual therapy sessions. This small pilot study (n = 16), which has recently been published [20], showed significant reductions in self-harming behavior, depressive symptoms and trait anxiety.

### Objectives

The aim of the study is to determine the effectiveness of the German CDP for treatment of NSSI in adolescents as compared to TAU. The main outcome objective of the trial is a clinically significant reduction (or remission) by at least 50% in the frequency of NSSI assessed by the Self-Injurious Thoughts and Behaviors Interview-German (SITBI-G) [21,22]. Secondary outcome criteria are a reduction in symptoms of depression as measured by the Beck Depression Inventory (BDI-II) [23] and an increase in self-reported well-being and self-esteem as measured by the KIDSCREEN [24] and Self-Esteem Scale (SES) [25], respectively. Additionally, comorbid psychiatric disorders and childhood adversity will be evaluated as predictors of therapeutic outcome.

### Primary hypothesis

Significantly more adolescents in the CDP group than in the TAU group will experience a clinically significant reduction in the frequency of non-suicidal self-injurious behaviors.

## Methods/Design

### Setting and recruitment

The trial is a single-center RCT performed at the Clinic of Child and Adolescent Psychiatry at the University Hospital of Heidelberg, Germany. The study protocol was approved by the ethics committee of the medical faculty at the University of Heidelberg. In all, 80 adolescents from across the geographical area of the city of Heidelberg between 12 and 17 years of age will be recruited. The potential participants and their families will be approached through our own in- and outpatient units, official notices posted in the surgical and the pediatric clinics, newspaper articles, advertisements on the clinic’s web page, and the study flyer. Adolescents will be initially screened via telephone to determine eligibility and to provide detailed information about the study.

### Inclusion and exclusion criteria

Boys and girls between 12 and 17 years of age who have engaged in at least five prior acts of cutting, burning, stabbing, hitting, excessive rubbing, and similar injuries during the past 6 months will be included. The last NSSI must have occurred within the month previous to the time of screening for the study. All adolescents are required to provide written informed consent to participate in the study, and for those under 16 years of age, written consent also must be provided by the parents/caregivers/legal substitute. Acute psychotic symptoms and acute intent to harm self or others, which requires immediate, intensive psychiatric intervention, constitute exclusion criteria. Other exclusion critieria are impaired intellectual functioning (according to clinical evaluation by a psychologist, whereby only adolescents who attend a regular school were assessed) and being in psychotherapy at the beginning of the study or already having completed NSSI therapy.

### Procedure and randomization

**Figure 1:**
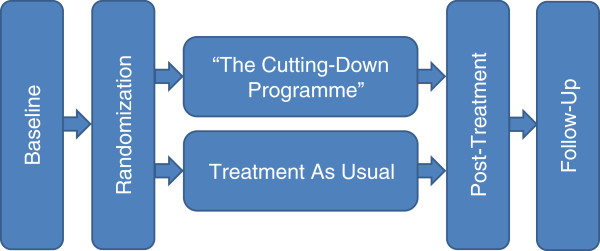
Overview of the trial procedure.

After obtaining informed and written consent according to the approved ethics committee’s protocol, an appointment for the baseline assessment will be set. The baseline assessment will take place at the Clinic of Child and Adolescent Psychiatry and will be performed by a clinical psychologist blinded to the group affiliation. In the baseline assessment, a broad range of psychological measures will be administered (see assessment). After eligibility is confirmed during baseline assessment, adolescents will be enrolled in the trial and will be randomly assigned to one of the two treatment groups as a baseline adaptive randomization. Each participant will be informed about the result of the randomization via telephone within seven days after their baseline assessment. Within the following days, the adolescents will receive a contact card, which is adapted according to the treatment group they are allocated to. Participants who are in the CDP group will promptly receive 8 to 12 weekly individual psychotherapy sessions. Participants in the TAU group will be referred to the public mental health care system. A special cooperation has been set up with the health care system to ensure that the participants will receive the first appointment there within the following 2 to 4 weeks. After completion of the CDP, the post-treatment assessment will be conducted immediately, which is approximately 4 months after baseline assessment. In order to match the assessment points between groups, TAU participants will be invited to their post-treatment assessment 4 months after the baseline. The follow-up assessment will take place 6 months after the post-treatment assessment for every participant. The timeframe of the trial is approximately 10 months for each of the participants, regardless of the treatment group. Figure 1 gives an overview of the trial procedure.

### Data assessment

Semi-structured interviews and self-report questionnaires will be conducted during the three assessments in this trial. The baseline assessment will take place before randomization and subsequent start of treatment.

For detailed assessment of NSSI, we will use the German version of the SITBI [22]. The SITBI-G is a semi-structured interview to assess the presence, frequency and characteristics of a wide range of self-injurious thoughts and behaviors, including suicidal ideation, suicide plans, suicide gestures, suicide attempts and NSSI [21]. The SITBI-G will be repeated at the post-treatment and follow-up assessment time points.

Furthermore, the baseline assessment comprises a sociodemographic interview, which will gather information about gender, date of birth, state of adolescents’ and parents’ education and current living situation. Comorbid diagnoses will be assessed using the German version of the Mini-International Neuropsychiatric Interview for children and adolescents (M.I.N.I- KID 6.0) [26] and parts of the Structured Clinical Interview for DSM-IV-Axis II (SCID-II) [27]. The M.I.N.I.-KID is a short, structured diagnostic interview for DSM-IV and ICD-10 psychiatric disorders for children and adolescents aged 6 to 17 years. The MINI-KID follows the structure format of the adult version of the interview. The MINI-KID is organized in diagnostic sections, each starting with screening questions for each disorder [26]. All questions are in a yes/no format. The SCID-II consists of a screening questionnaire and a subsequent interview to assess axis II disorders. The questionnaire consists of 117 screening questions for the twelve personality disorders. In the interview only questions answered ‘yes’ require further assessment [27]. In our trial the following parts of the SCID-II interview will be used: avoidant, dependent, borderline and antisocial personality disorder. Traumatic and adverse childhood experiences will be assessed using the German version of the Childhood Experience of Care and Abuse (CECA) interview, which is a semi-structured, retrospective interview [28,29]. The CECA includes detailed assessment of the following types of adversity: loss of a parent, parental neglect and antipathy, physical, sexual and psychological abuse, family arrangements, discord at home, violence between parents, supervision and control of children, role reversal and childhood helplessness. In addition to these adverse experiences, the CECA contains the positive scales: support, closeness to parents, coping and being the parents’ favorite child [28,29]. The sociobiographic history, the M.I.N.I-KID, the CECA and the SCID-II interviews are parts of the baseline assessment and will not be repeated at post-treatment or follow-up.

Three questionnaires need to be completed during the assessment: The German version of the Beck Depression Inventory II (BDI-II) will be used to assess the intensity of depression according to the DSM-IV criteria. It consists of 21 items. Each item is a list of four statements arranged in increasing severity about a particular symptom of depression. The time frame for the response is two weeks. A value of 0 to 3 is assigned for each answer. A total score can be summed up. A score of 0 to 13 indicates minimal depression, 14 to 19 indicates mild depression, 20 to 28 indicates moderate depression and a score 29 to 63 indicates severe depression. A higher total score indicates more severe depressive symptoms. The BDI-II is widely used and is considered reliable (r = .93) and valid (r = .32-68) [23]. The KIDSCREEN questionnaire assesses adolescents’ subjective health and well-being [24]. It is normed for children and adolescent aged between 8 to 18 years. There are three KIDSCREEN instruments available for children and adolescents as well as parent/proxy versions. In our trial we will use the KIDSCREEN-27 which consists of five Rasch-scaled dimensions: physical well-being, psychological well-being, autonomy and parents, peers and social support and school environment. This version is widely used and shows good reliability (α = .70) and validity (r = .56) [24]. The Rosenberg Self-Esteem Scale (SES) is a 10-item self-report measure of global self-esteem. It consists of 10 statements related to overall feelings of self-worth or self-acceptance. The items are answered on a four-point scale ranging from ‘strongly agree’ to ‘strongly disagree’ [25]. The BDI-II, the KIDSCREEN and the SES questionnaires will be repeated at the post-treatment and follow-up assessments.

The *Fragebogen zur Patientenzufriedenheit* (ZUF-8; Patient Satisfaction Questionnaire) is a German questionnaire to assess treatment satisfaction of patients. It consists of eight statements, which are answered on a four-point scale ranging from ‘very satisfied’ to ‘quite dissatisfied’ [30]. The ZUF will be part of the post-treatment and the follow-up assessment.

Table 1 gives a summary of all measures used and the corresponding time of assessment.

**Table 1:** Measures and time of assessment

**Measure**	**Baseline**	**Post-treatment**	**Follow-up**
SITBI-G	X	X	X
M.I.N.I.-Kid	X		
SCID-II	X		
CECA	X		
BDI-II	X	X	X
SES	X	X	X
KIDSCREEN	X	X	X
ZUF-8	X	X	X

*BDI-II* Beck Depression Inventory II, *CECA* Childhood Experience of Care and Abuse interview, *KIDSCREEN* Health Related Quality of Life Questionnaire for Children and Young People, *M.I.N.I- KID* Mini-International Neuropsychiatric Interview for children and adolescents, *SES* Rosenberg Self-Esteem Scale, *SCID-II* Structured Clinical Interview for DSM-IV-Axis II, *SITBI-G* Self-Injurious Thoughts and Behaviors Interview-German, *ZUF-8* Patient Satisfaction Questionnaire.

### Participants’ incentive

All participating adolescents will receive 50 Euro for each of the three assessments.

### Intervention

Each participant will be randomized to one of the two possible treatment conditions (CDP versus TAU). At the end of the trial it is assumed that 40 randomly selected participants will be in each intervention condition.

### Intervention one

Participants will be informed about being allocated to the TAU group via telephone by a trained research psychologist. During this telephone call, they will be encouraged to seek treatment and will be referred to the existing mental health care system. They receive contact details of established child psychotherapists, psychiatrists, clinics or counseling centers. The child psychotherapists, psychiatrists, clinics and counseling center have all been informed about the study and have agreed to take part in it. This collaboration was established to shorten the waiting times (first appointment within 2 to 4 weeks) and to guarantee rapid access to treatment for all adolescents. In addition to the referral, each participant will receive a contact card including the study center's telephone number and the emergency number of our clinic. This card ensures that all participants can contact our psychological staff members if they have any questions or problems at any time, and that participants receive adequate crisis intervention if needed.

### Intervention two

CDP is a manualized, short-term psychotherapy which is designed for 8 to 12 therapeutic sessions. It includes elements of cognitive-behavioral therapy (CBT) and dialectic-behavioral therapy (DBT) and is specifically tailored to the treatment of self-injurious behavior in adolescents. The content of the treatment is structured in a manual for patients and a separate manual for therapists. The modules in the manual were all developed from a comprehensive literature review on the treatment of DSH including associated psychological factors performed by the English authors who originally developed the manual [20]. The program comprises four main modules, which can be individually expanded by optional modules - according to requirements of the patient. The four main modules are promoting therapy motivation and compliance of the subjects, identifying reasons for the existing behavior, testing alternative behaviors instead of NSSI, and stabilizing alternative behaviors to reduce aversive strains, which often activate NSSI. In addition to individual therapy sessions, the participating adolescents will be encouraged to do certain homework, which facilitates preparation and post-processing of the therapy sessions in order to increase their effectiveness. Like the participants in the TAU group, the participants in the CDP will receive a contact card so that they can contact one of our psychological staff members if they have any questions or problems at any time and get access to the clinic's crisis response system.

### Staff

During the trial the study assessments will be performed by a clinical psychologist. It is important to note that data collection and psychotherapy are strictly separated in this trial. The assessments will always be performed by the same clinical psychologist, who is blinded to the randomization and the respective treatment group. The psychotherapists giving treatments are kept unaware of the results of the psychometric assessments.

### Training and support of the staff members

All treating psychotherapists in the CDP will be supervised on a monthly basis by a specifically trained supervisor (with a clinical background in CBT and DBT) who will ensure quality and adherence to the treatment manual. To guarantee good adherence, the therapist documents all sessions in according protocols and, if the participants have agreed, records the session. The protocols are checked every week. In emergency situations, all clients have unlimited access to the on-call system of the Clinic of Child and Adolescent Psychiatry.

### Sample size

With an expected response rate of 25% in TAU and 60% in CDP, a sample size of 40 for each group is needed to achieve sufficient power (85%).

### Statistical analysis

To ensure objectivity of the data collection setting the whole process has been standardized. Data collection and treatment will be strictly separated and performed by different personnel (blinded). The data collection process comprises the baseline, the post-treatment and the follow-up assessment. All analyses will be intent-to-treat analyses. If post-treatment or follow-up assessments are missing, the missing assessment will be filled with the last non-missing value. NSSI, which is the main outcome of this trial, will be assessed using the SITBI-G. A clinically significant improvement is defined as a reduction in the frequency of NSSI by 50%. In this process the TAU group and the CDP group will be compared with each other to determine any differences in primary and secondary outcomes by using a *χ*^2^ - test. As a secondary measure, ANCOVA with group as factor and frequency of NSSI at baseline as covariate will be assessed. Explorative analysis will be executed to account for the influence of childhood trauma, psychiatric diagnosis and personality traits and other factors potentially influencing the patients (for example, gender). Descriptive analysis will be used to characterize the study sample. For nominal data frequencies will be determined, and for continuous data means and standard deviations with confidence intervals will be calculated. A significance level of α = .05 will be chosen. Statistical analysis will be performed using Stata 13.

### Ethical issues

The study will be performed in accordance with the Declaration of Helsinki. Approval for the study was obtained from the ethics commission of the Faculty of Medicine at the University of Heidelberg (Ethics Committee No.: S-363/2011). Only those who have provided written informed consent will be included in the trial. If the participants are younger than 16, caregivers’ written consent must be provided as well. Each potential participant and his/her family will be contacted by telephone and provided with a copy of the information sheets, which include a contact number, so that they have the opportunity to ask questions.

## Discussion

This present study in adolescents with NSSI is the first RCT worldwide that uses the CDP. We are testing the brief therapeutic intervention program to help adolescents who show repetitive NSSI, some of whom have not received any treatment for years. The aim of this treatment program is to end, or at least significantly reduce NSSI. The use of a short-term psychotherapeutic intervention may have many more benefits for these adolescents and their families. For example, adolescents may learn how to better handle their feelings and acquire strategies for a less stressful everyday life, especially with regard to family life.

The potential benefit of the ‘The Cutting-Down Programme’ has recently been shown in the study published by Taylor *et al*. [20]. This study found significant reductions in self-harming behavior, depression symptoms and trait anxiety; however, no control group was used to account for non-specific study effects [20].

In Germany this is the first trial to test a short-term therapy in adolescents engaging in NSSI. It is very important to note that this short-term therapy constitutes a very specific approach with a low threshold because of its short time frame. That should help to increase motivation to access therapy and decrease the fear of being stigmatized or stigmatizing themselves.

To the best of our knowledge, no other such specific treatment for treating young people with NSSI exists worldwide, although prevalence of NSSI is high and has continued to rise within the last year [31]. Most of the approaches for treating NSSI are part of the DBT-A program, which has been primarily designed for patients with BPD and for which evidence for use in adolescents is lacking. However, treating all adolescents engaging in NSSI with a BPD-specific program may not be the best approach, considering financial resources available in many public mental health systems. It is known that the existing programs do not always stop or reduce NSSI. Moreover, many of the adolescents engaging in NSSI do not fulfill the criteria of any psychiatric diagnosis [31]; therefore, a specific program and diagnosis is needed for NSSI itself. In our opinion this new approach may be a better way to encourage young people with NSSI to get necessary and specific treatment, which may be sufficient for termination of NSSI within a short period of time. We propose that the program is very effective in terms of reducing or terminating NSSI and it will not involve high economic costs, especially because it is a short-term program.

### Limitations

The Heidelberg region is a particularly wealthy part of Germany, which may have the effect that our findings cannot be generalized to more rural or socioeconomically poorer areas. However, we aim to include every interested adolescent who fits the inclusion criteria without any regard for background (for example, adolescents who live in assisted living, a children’s home or a more rural region around Heidelberg).

If the program proves to be clinically effective, it can be delivered widely across psychologists, psychiatrists and therapists who deal with NSSI in adolescents.

## Trial status

Baseline assessments and subsequent intervention started in November 2012 and so far 22 participants have been randomized. The entire trial is scheduled to be completed by the end of 2014.

## Abbreviations

BDI-II: Beck Depression Inventory; BPD: Borderline personality disorder; CECA: Childhood Experience of Care and Abuse interview; CDP: The Cutting-Down-Programme; DBT-A: Dialectic behavioral therapy for adolescents; DSH: Deliberate self-harm; DSM-IV: Diagnostic and Statistical Manual of Mental Disorders-IV; ICD-10: International Statistical Classification of Diseases and Related Health Problems-10; KIDSCREEN: Health Related Quality of Life Questionnaire for Children and Young People; MACT: Manual-Assisted Cognitive-behavioural Therapy; MBT: Mentalization-based therapy; M.I.N.I- KID: Mini-International Neuropsychiatric Interview for children and adolescents; NSSI: Non-suicidal self-injury; RCT: Randomized controlled trial; SES: Self-Esteem Scale; SCID-II: Structured Clinical Interview for DSM-IV-Axis II; SITBI-G: Self-Injurious Thoughts and Behaviors Interview-German; TAU: Treatment as usual; ZUF-8: Patient Satisfaction Questionnaire.

## Competing interests

The authors declare that they have no competing interests.

## Authors’ contributions

GF participated in the design and the coordination of the study and wrote the first draft of the manuscript. PP participated in the design of the study, performed the statistical analysis and revised the article critically. RB and FR participated in the design of the study and revised the article critically. MK was responsible for the design and coordination of the study, and helped to draft the manuscript. All authors read and approved the final manuscript.

## References

[B1] Lloyd-Richardson EE Perrine N Dierker L Kelley ML Characteristics and functions of non-suicidal self-injury in a community sample of adolescents Psychol Med 2007 37 1183 1192 10.1017/S003329170700027X 17349105 PMC2538378

[B2] Whitlock J Knox KL The relationship between self-injurious behavior and suicide in a young adult population Arch Pediatr Adolesc Med 2007 161 634 640 10.1001/archpedi.161.7.634 17606825

[B3] Ross S Heath N A Study of the Frequency of Self-Mutilation in a Community Sample of Adolescents J Youth Adolesc 2002 31 67 77 10.1023/A:1014089117419

[B4] Plener PL Libal G Keller F Fegert JM Muehlenkamp JJ An international comparison of adolescent non-suicidal self-injury (NSSI) and suicide attempts: Germany and the USA Psychol Med 2009 39 1549 1558 10.1017/S0033291708005114 19171079

[B5] Brunner R Parzer P Haffner J Steen R Roos J Klett M Resch F Prevalence and psychological correlates of occasional and repetitive deliberate self-harm in adolescents Arch Pediatr Adolesc Med 2007 161 641 649 10.1001/archpedi.161.7.641 17606826

[B6] Fischer G Göbelbecker L Schneider S Seyle Ergebnisbericht 2011 Heidelberg: Klinik für Kinder- und Jugendpsychiatrie, Universitätsklinikum Heidelberg

[B7] Nock MK Joiner TE Jr Gordon KH Lloyd-Richardson E Prinstein MJ Non-suicidal self-injury among adolescents: diagnostic correlates and relation to suicide attempts Psychiatry Res 2006 144 65 72 10.1016/j.psychres.2006.05.010 16887199

[B8] Jacobson CM Muehlenkamp JJ Miller AL Turner JB Psychiatric impairment among adolescents engaging in different types of deliberate self-harm J Clin Child Adolesc Psychol 2008 37 363 375 10.1080/15374410801955771 18470773

[B9] Hawton K Harriss L Deliberate self-harm in young people: characteristics and subsequent mortality in a 20-year cohort of patients presenting to hospital J Clin Psychiatry 2007 68 1574 1583 10.4088/JCP.v68n1017 17960975

[B10] Plener PL Kapusta ND Kölch MG Kaess M Brunner R Nicht-suizidale Selbstverletzung als eigenständige Diagnose. Implikationen des DSM-5 Vorschlages für Forschung und Klinik selbstverletzenden Verhaltens bei Jugendlichen Z Für Kinder Jugendpsychiatrie Psychother 2012 40 113 120 10.1024/1422-4917/a000158 22354495

[B11] American Psychiatric Association Diagnostic and Statistical Manual of Mental Disorders, Fifth Edition (Dsm-5) 2013 Arlington, VA, US: American Psychiatric Publishing

[B12] Plener PL Fegert JM Non-suicidal self-injury: state of the art perspective of a proposed new syndrome for DSM V Child Adolesc Psychiatry Ment Health 2012 6 9 10.1186/1753-2000-6-9 22463601 PMC3350395

[B13] Bohme R Fleischhaker C Mayer-Bruns F Schulz E Arbeitsbuch. Dialektisch- Behaviorale Therapie für Jugendliche (DBT-A) – Therapiemanual 2007 Hauptstr. 8, 79104 Freiburg, Germany: University of Freiburg, Department for Child and Adolescent Psychiatry and Psychotherapy

[B14] Foelsch PA Odom AE Schmeck K Schlüter-Müller S Kernberg OF Behandlung von Adoleszenten mit Identitätsdiffusion - Eine Modifikation der Übertragungsfokussierten Psychotherapie (TFP) Persönlichkeitsstörungen Theor Ther 2008 12 153 16224052085

[B15] Rathus JH Miller AL Dialectical behavior therapy adapted for suicidal adolescents Suicide Life Threat Behav 2002 32 146 157 10.1521/suli.32.2.146.24399 12079031

[B16] Wilkinson P Goodyer I Non-suicidal self-inj Eur Child Adolesc Psychiatry 2011 20 103 108 10.1007/s00787-010-0156-y 21222215

[B17] Rossouw TI Fonagy P Mentalization-based treatment for self-harm in adolescents: a randomized controlled trial J Am Acad Child Adolesc Psychiatry 2012 51 1304 1313 10.1016/j.jaac.2012.09.018 23200287

[B18] Kaess M Selbstverletzendes Verhalten: Entwicklungsrisiken erkennen und behandeln 2012 Beltz Psychologie Verlags Union: Weinheim

[B19] Evans K Tyrer P Catalan J Schmidt U Davidson K Dent J Tata P Thornton S Barber J Thompson S Manual-assisted cognitive-behaviour therapy (MACT): a randomized controlled trial of a brief intervention with bibliotherapy in the treatment of recurrent deliberate self-harm Psychol Med 1999 29 19 25 10.1017/S003329179800765X 10077290

[B20] Taylor LMW Oldershaw A Richards C Davidson K Schmidt U Simic M Development and pilot evaluation of a manualized cognitive-behavioural treatment package for adolescent self-harm Behav Cogn Psychother 2011 39 619 625 10.1017/S1352465811000075 21392417

[B21] Nock MK Holmberg EB Photos VI Michel BD Self-Injurious Thoughts and Behaviors Interview: development, reliability, and validity in an adolescent sample Psychol Assess 2007 19 309 317 17845122 10.1037/1040-3590.19.3.309

[B22] Plener PL Fischer G Groschwitz RC Kaess M Das Self-Injurious Thoughts and Behaviors Interview- German 2012 Steinhövelstrasse 5, 89075 Ulm, Germany: Department of Child and Adolescent Psychiatry and Psychotherapy, University of Ulm

[B23] Beck AT Steer RA Brown GK Beck-Depressions-Inventar (2. Auflage) 2006 Frankfurt/M: Harcourt Test Service GmbH

[B24] Ravens-Sieberer U Gosch A Erhart M von Rueden U The KIDSCREEN Group Europe: The KIDSCREEN Questionnaires 2006 1 Lengerich: Pabst Science Publisher

[B25] Rosenberg M Society and the adolescent self-image 1989 Princeton, NJ: Princeton University Press

[B26] Sheehan DV Lecrubier Y Sheehan KH Amorim P Janavs J Weiller E Hergueta T Baker R Dunbar GC The Mini-International Neuropsychiatric Interview (M.I.N.I.): the development and validation of a structured diagnostic psychiatric interview for DSM-IV and ICD-10 J Clin Psychiatry 1998 59 20 22 33 9881538

[B27] Fydrich T Renneberg B Schmitz B Wittchen H-U SKID-II - Strukturiertes Klinisches Interview Für DSM-IV, Achse II: Persönlichkeitsstörungen 1997 Göttingen: Hogrefe

[B28] Bifulco A Brown GW Harris TO Childhood Experience of Care and Abuse (CECA): a retrospective interview measure J Child Psychol Psychiatry 1994 35 1419 1435 10.1111/j.1469-7610.1994.tb01284.x 7868637

[B29] Kaess M Parzer P Mattern M Resch F Bifulco A Brunner R Childhood Experiences of Care and Abuse (CECA): Validierung der deutschen Version von Fragebogen und korrespondierendem Interview sowie Ergebnisse einer Untersuchung von Zusammenhängen belastender Kindheitserlebnisse mit suizidalen Verhaltensweisen Z Für Kinder Jugendpsychiatr Psychother 2011 39 243 252 10.1024/1422-4917/a000115 21667449

[B30] Schmidt J Lamprecht F Wittmann WW Zufriedenheit mit der stationären Versorgung. Entwicklung eines Fragebogens und erste Validitätsuntersuchungen Psychother Psychosom Med Psychol 1989 39 248 255 2762479

[B31] Wilkinson P Non-suicidal self-injury Eur Child Adolesc Psychiatry 2013 22 Suppl 1 S75 S79 23202887 10.1007/s00787-012-0365-7

